# A Feasibility Study for an Integrated Approach to Fall Prevention in Community Care: Stay Up and Active in Orange County

**DOI:** 10.3389/fpubh.2016.00174

**Published:** 2016-08-29

**Authors:** Spencer W. Lindgren, Katie Kwaschyn, Ellen Roberts, Jan Busby-Whitehead, Lori A. Evarts, Tiffany Shubert

**Affiliations:** ^1^Orange County Emergency Services, University of North Carolina at Chapel Hill, Chapel Hill, NC, USA; ^2^University of North Carolina at Chapel Hill, Chapel Hill, NC, USA; ^3^Division of Geriatric Medicine, University of North Carolina at Chapel Hill, Chapel Hill, NC, USA; ^4^Public Health Leadership Program, Gillings School of Global Public Health, University of North Carolina at Chapel Hill, Chapel Hill, NC, USA; ^5^Center for Aging and Health, University of North Carolina at Chapel Hill, Chapel Hill, NC, USA

**Keywords:** first responders, fall prevention program, aging and longetivity, emergency medical services, STEADI toolkit

## Abstract

**Introduction:**

Falls among persons over 60 present significant risks for serious injury or debility. Falls place burdens on Emergency Medical Services (EMS), hospitals, and the adults themselves. Recognizing a need to provide interventions to minimize risk, Orange County Emergency Services (OCES), the Orange County Department on Aging (OCDoA), and the University of North Carolina at Chapel Hill (UNC) partnered to create the Stay Up and Active Program (SUAA). The purpose of this study was to determine if SUAA was a feasible program to implement in the community.

**Methods:**

A streamlined workflow algorithm between the OCES and OCDoA was created and employed to provide falls risk assessment and necessary services. Qualitative techniques were used to assess the need for such a program and its potential impact. A subset of individuals was interviewed 3 months after the intervention to assess the impact of the intervention on their fall risk. Formal stakeholder interviews were not conducted, but anecdotal information from EMS providers was obtained and reported.

**Results:**

In the first 7 months, 478 instances of individuals who called OCES screened positive for falls risk. Of the 478 positive screenings, 55 individuals were identified as having received more than one positive fall screen due to multiple calls. The maximum number of positive screenings by one individual was 14. More women (61.3%) than men screened positive for fall risk. Individuals 88 years of age (6.9%) represented the highest number of individuals with positive screens. Nineteen (4.0%) people who called OCES and received the intervention completed a 3-month follow-up survey. Of the 19, 86% (*n* = 16) reported no recurrent fall.

**Conclusion:**

The number of individuals who screened positive supports the need for early identification and intervention through SUAA. This program identified several challenges connecting older adults with services already available to keep them independent, which provided insight to all stakeholders regarding factors that inhibit the program’s success. The program evaluation should continue to provide suggestions for improvement and ensure sustainability.

## Introduction

Falls in older adults comprise a significant portion of health-care expenditures and resource use in the United States. One of every three older adults falls annually resulting in a total of 12 million falls (1). In 2013, these falls represented approximately $34 billion in direct medical costs and led to 21,700 deaths among older adults (2).

North Carolina is ranked fifth in the United States for the greatest number of older adults. It is projected that by 2030, there will be a 32% increase in the state’s population aged 65 years and older (3). Given these demographics, the state is particularly concerned by this public health issue, keeping in mind that the costs of falls and the burden on the health-care system are already substantial. In 2012, there were nearly 195,000 Emergency Department (ED) visits as a result of unintentional falls (4). Of these ED visits, 900 resulted in deaths, constituting a 74.5% increase in deaths from falls between 1999 and 2012 (4).

Orange County is one of the 100 counties in North Carolina. The county measures approximately 398 square miles and is home to 141,354 citizens (5). The county is home to several towns including Chapel Hill, Carrboro, and Hillsborough. The University of North Carolina at Chapel Hill (UNC), along with the UNC Hospitals System, are located in the southern part of the county in a more urban setting, while the northern part of the county is mostly rural with a significantly lower population density. The area has gained popularity with retirees and is the home of five large retirement communities as well as several assisted living and skilled nursing facilities. Persons age 65 years or older make up 11.2% of the population and it is projected that by 2030 18% of the population will be 65 years or older (3, 5). Females comprise 52.2% of the population, Caucasians account for 76.8% of the population, African-American for 12.2%, Hispanic for 8.4%, Asian for 7.7%, and Native American for 0.6% (5).

The Emergency Medical Services Division (EMS) of the Orange County Emergency Services (OCES) is the sole provider of Advanced Life Support (ALS) services in the county. EMS consists of 75 full-time and 20 part-time employees, and staffs 5–9 ambulances any given day. OCES began tracking EMS calls classified as falls-related in 2010. Between 2010 and 2013, the EMS Division averaged 10,384 calls per year (6). Of these calls, 0.9% were lift assist EMS calls (where a person needs help transferring from a bed to a chair or similar situation and has not actually fallen), and 10.7% were falls-related calls, for a combined average of 11.6% of all calls being falls-related over the 4-year period; these data are summarized in Table 1 (6).

**Table 1:** **Baseline falls data for Orange County emergency medical services (EMS), 2010–2013 (6)**.

Year	Total EMS calls	Lifting assistance	Falls	Combined lifting and falls	Percent lifting and falls
2010	9,585	159	984	1,143	11.9
2011	10,333	101	1,117	1,218	11.8
2012	10,636	64	1,165	1,229	11.6
2013	10,983	63	1,182	1,245	11.3

*During the period of 2010–2013, there was no tracking to identify repeat fall victims that utilized an EMS ambulance for transport or non-transport purposes*.

The Orange County Department on Aging (OCDoA) encounters over 190,000 participants per year at its hosted events (7). The OCDoA has expertise and resources to help older adults manage their fall risk and achieve the goals of aging in community. The Aging Transitions Unit, a group within OCDoA, employs five full-time and several part-time employees to provide in-home assessments, caregiver referral, low-cost support services, and other age-related services to citizens (7). The Aging Transitions Unit spends an average of 150 h per month providing information and case assistance to citizens (7).

Emergency Medical Services has frequent contact with older adults who would benefit from OCDoA services to minimize their risk of falling. The opportunity to leverage the “first responder” relationship and connect older, at-risk adults with the resources in the community was the inspiration for the Stay Up and Active Program (SUAA). SUAA was designed to be a fall risk identification and management program implemented by EMS to connect at-risk older adults with the services they need. The intent of connecting these adults with services is to reduce the number of falls by older adults in their homes. This program represents the first time EMS formally collaborated with the Department on Aging to meet a need in the community.

Initial discussions between the OCDoA and EMS supported that an EMS-centric model would be optimal for a community falls prevention program. As EMS providers are frequently the first caregivers in any fall, OCES was well positioned to link the at-risk population with the services and resources provided by the OCDoA. EMS providers have the opportunity to obtain accurate and complete histories from the older adults and possible bystanders on scene, and can assess the older adult’s safety in their environment. EMS and OCDoA agreed to initiate SUAA with EMS as the first point of contact with potential at-risk individuals. EMS would then schedule an in-home visit to further assess the older adult and communicate with OCDoA to connect the older adults with community resources.

The purpose of this study was to determine if SUAA was a feasible program to implement in the community. Specifically, it was necessary to know if the perceived need for the program was accurate, if the workflow developed to implement SUAA for EMS staff was efficient and effective, if older adults who called EMS for a falls-related issue would be receptive to a second home visit, and if the system designed to facilitate communication between OCES and OCDoA achieved the goals of the project. The information from this study will help inform future steps to this collaborative project to address the problem of falls in the community.

## Materials and Methods

### Development of Workflow

**Figure 1:**
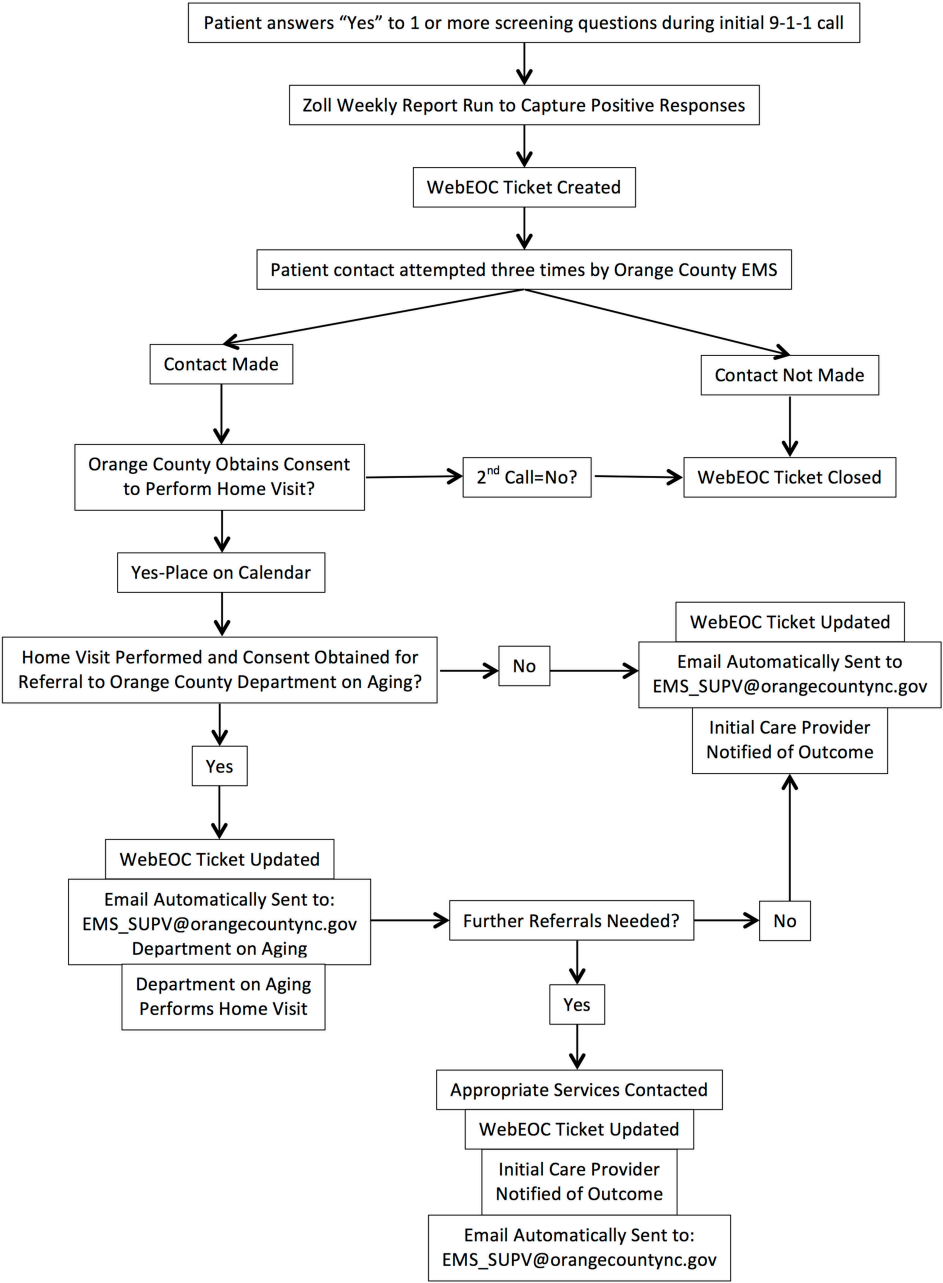
**WebEOC flow chart**.

The OCDoA provides services to all county residents aged 60 years and older. Therefore, the SUAA program included any adult who is 60 years of age or older in Orange County who received EMS support resulting from a call for service. An algorithm and workflow were developed by both organizations to identify the level of risk and appropriate intervention (Figure 1).

All adults over the age of 60 years, who called EMS, were screened for fall risk. During the course of an EMS intervention, providers would ask the following screening questions from the American Geriatrics Society (AGS) Clinical Practice Guidelines questions:

• Are you worried that you are going to fall?
• Have you fallen in the past year?
• Are you unsteady when walking or standing? (8)

A positive screen was based on a “Yes” response to any of the questions. Those that screened positive were asked if they would like to receive a follow-up phone call and additional home safety services from EMS. Those who agreed signed a form allowing EMS to access their name and phone number for further follow-up. Any EMS provider, regardless of their certification level, was able to conduct a fall risk screen. This screening was designed to supplement the standard patient assessment, and was incorporated to be as streamlined as possible for field EMS staff.

The older adults who agreed to follow-up were entered into the EMS WebEOC (online emergency incident management technology) database to track their status. The purpose of WebEOC was to notify the SUAA team that an older adult screened positive for the program, to track their status and to communicate outcomes between agencies. Seventy-two hours following the initial EMS service call, a follow-up telephone call by EMS was initiated to schedule a home visit. If no contact was made after three telephone call attempts, the patient record was closed. If contact was made, a home visit from EMS personnel would be scheduled.

The home visit consisted of a translation of the Centers for Disease Control’s STEADI (Stopping Elderly Accidents, Deaths, and Injuries) toolkit, which is an evidence-based fall risk management algorithm for clinicians (9). The STEADI algorithm includes assessment of the following risk factors: falls history, fear of falling, polypharmacy, leg weakness, balance impairments, low vision, cognitive impairment, depression as well as environmental factors. At the scheduled EMS home visit, the patient would be asked background information including current medications, medication history, and the current status of their health. Additionally, they would be screened with validated tools for cognitive impairment (Mini-Cog Assessment), depression (PHQ-2), elder abuse, and vision impairment (9). The older adult completed a Timed Up and Go Test, the 30-S Chair Stand, and 4-Stage Balance Test Full Tandem Stance (9). Finally, an assessment of their current living conditions and any observed safety concerns or risk factors were discussed with the patient. The results of the EMS home visit were then entered into WebEOC.

Subsequent to a home visit from EMS, and with approval from the older adult, a notification was sent to OCDoA from the WebEOC database. The goal was for OCDoA to make an assessment and connect the older adult with the appropriate resources in the community. At the OCDoA follow-up, appropriate referrals for occupational therapy, physical therapy, counseling, caregiver support group, in-home health-care services, and others were made.

### Communication

In an effort to streamline communication and share findings, the assessments and referrals were recorded in WebEOC by both EMS and by OCDoA and used for participant monitoring. Following the completion of a WebEOC ticket, the initial EMS crews were notified of the outcome of OCES and OCDoA follow-up with the older adults.

Prior to implementation, SUAA was reviewed by the UNC Office of Human Research Ethics Institutional Review Board as Study 13-2942 and was granted exempt status from further review as the submission was considered a quality improvement program and did not constitute human subjects research under 45 CFR 46.102 (d or f) and 21 CFR 56.102(c)(e)(l). Additionally, neither special funding was allocated nor was grant funding obtained to implement this program. All resources were obtained from preexisting sources within county offices. Any materials given to adult participants were free of charge and donated by relevant organizations.

### Evaluation

The SUAA program was evaluated by a team based at the UNC Chapel Hill Center for Aging and Health to determine the feasibility of implementing the project. The first part of the evaluation determined the SUAA program met a need in the community. Implementation based on the established workflow and related IRB status enabled the data collection and analysis effort. The second part of the SUAA program evaluation consisted of interviews with a subset of individuals who received the home visit to assess their response to the program. The evaluation work was funded by a grant received by the University of North Carolina.

Formal interviews of EMS providers were not conducted; however, anecdotal information obtained by providers was obtained when reviewing cases with these providers. The primary method of obtaining information about patients and their condition was by reviewing the patient care reports submitted by EMS providers after the initial 9-1-1 call. These reports provided valuable information about the patient’s health and current social environment.

## Results

**Figure 2:**
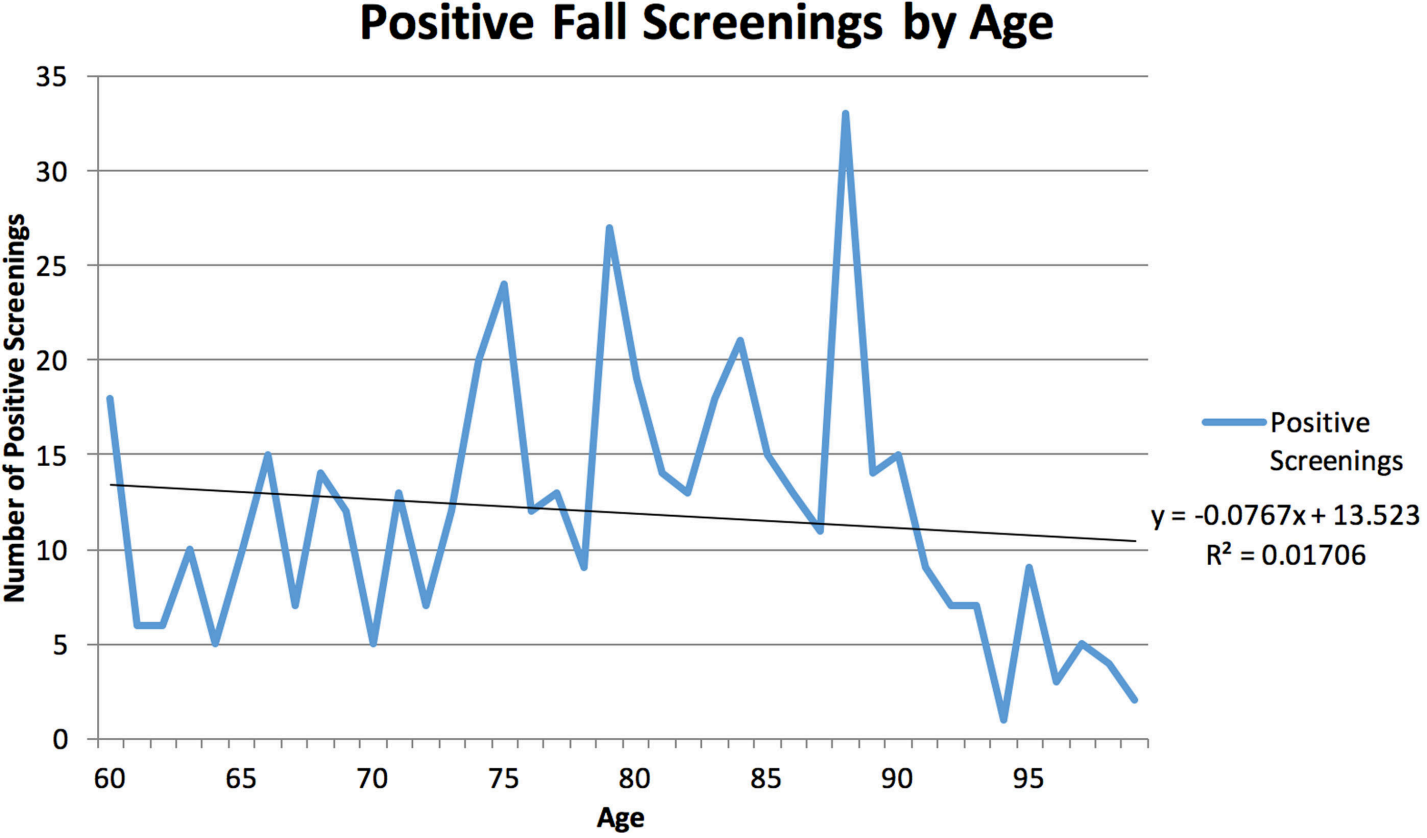
**Positive fall screenings by patient age, Orange County, NC, USA, September 1, 2013–March 31, 2014**.

Between September 1, 2013 and March 31, 2014, there were a total of 704 EMS calls for a fall and 37 EMS calls for “Lift Assistance” (6). There were a total of 478 instances of a positive screen using the Falls Risk Assessment criteria. Of these, there were a total of 55 unique individuals who had at least one repeat positive screen as a result of a 9-1-1 call. The range of repeat screenings by an individual was between two and fourteen. There were 32 individuals who experienced a total of two positive screenings and 303 individuals with only one positive screening. The available data are presented in Table 2. The age range of patients was 60–99 years, with the largest group (6.9%) being adults aged 88 years. Females made up 61.3% of the positive screenings. Positive screenings are plotted by age of the patient in Figure 2. The age demographics of Orange County EMS patients decreases significantly after age 90 years, accounting for the drop in positive fall screenings. Of the patients who screened positive for fall risk, 316 instances (66.1%) were transported to an ED, and the remaining 162 instances (33.9%) were non-transport either by Refusal Against Medical Advice or Referral to a Physician within 4 or 24 h.

**Table 2:** **Positive screening rates for September 1, 2013 through March 31, 2014 (6)**.

Total number of positive screenings	Number of individuals
1	303
2	32
3	10
4	5
5	2
6	1
7	2
8	1
9	1
10	0
11	0
12	0
13	0
14	1

Of patients who screened positive, accurate phone numbers were only recorded in 31% (148 instances) of the patient care reports. Of the 148 instances of positive screening for falls risk and accurate phone number collected, 54 participants agreed to a home visit by EMS. Of those patients who received a home visit by EMS, 20 participants agreed to a follow-up visit from the OCDoA. Nineteen of the participants who received follow-up from the OCDoA agreed to further follow-up from UNC in the form of a 3-month follow-up survey conducted by phone interview.

Of the 19 participants who completed the 3-month follow-up survey, 86% did not report a recurrent fall at 3-month follow-up. A total of 74% were very satisfied, and 26% were satisfied with the home visit from EMS. When asked about the value of the program, 5% found it not helpful, 16% found it somewhat helpful, 21% found it helpful, and 42% very helpful; 10 respondents answered other. As a result of the home visit, 16% felt somewhat confident, 16% felt confident, and 32% felt very confident that he or she could take actions to reduce risk of falling. Ten participants remarked other and commented, “They have already done everything they could to prevent falls” and, “The visit helped to heighten their awareness that they had to do something to prevent falls.” In the survey, 95% would recommend the EMS home visit program to a friend who may need help to stay independent in their home and 5% responded maybe.

## Discussion

Older adult falls, and older adults who fall more than once, are a public health problem in Orange County, NC, USA. With more than 11% of all EMS calls being fall-related, coupled with a rapidly expanding aging population within the community, there was a definite need for a local structured falls prevention program. An efficient workflow incorporating evidence-based assessments was constructed and adopted by OCES and OCDoA. The WebEOC system allowed for transparent and timely exchanges of information between providers. The program recruited only 54 older adults in a 7-month pilot period, a lower than expected number. Of the 19 participants who completed the 3-month follow-up survey, 86% did not report a recurrent fall and the overall satisfaction rate was positive.

During the 7-month trial period, there were a total of 704 EMS calls for a fall, and 37 EMS calls for “Lift Assistance.” Based on this information alone, the EMS unit hour utilization and ED bed time use expected as a result of these calls causes a significant burden to health-care resources. Further studies are warranted to investigate if SUAA has any impact on decreasing the number of annual falls related EMS calls. First responders should continue to be utilized as they offer a unique and innovative way to access older, at-risk adults who would otherwise be left underserved by their community resources.

There were a total of 741 falls related EMS calls during the study period, but only 478 instances of positive screenings. As the falls risk assessment could be performed on any patient aged 60 years or greater no matter what the nature of the call (Fall, Lift Assist, Chest Pain, Dyspnea, etc.), it was expected that at least as many positive screenings would be recorded. Since this is not the case, further investigation is needed to determine and quantify whether or not all fall victims were screened or if they screened negative. If they are simply were not being screened, then further training and emphasis will need to be placed on the necessity for asking the three falls risk assessment questions with field EMS staff. If the patients are screening negative, then evaluation of EMS recording and other potential areas of outreach need to be explored with this program.

In examining the demographics of the patients who screened positive, more women screened positive than men, consistent with the national data that shows women over 60 falls more frequently than men (1). The Orange County data do show, however, that there is no correlation between age and a positive falls screening. The most common age in Orange County for falls risk was 88 years, but otherwise, there was no ability to predict a person’s risk based on age. EMS providers in Orange County anecdotally believe that more falls calls occur at assisted living facilities than at private residences. Based on the screenings performed by EMS, these data revealed that there were, in fact, more people at risk for falling in private homes than in assisted living facilities.

There were several barriers and limitations discovered during implementation of this program. Barriers fell into two categories: system-based change and older adults. Initially, information sharing to track participants who agreed to follow-up was difficult. In response, the WebEOC boards were reviewed, modified, and republished to ensure ease of access and use for all agencies. A second barrier encountered was the poor phone number collection by field EMS staff. Without an accurate phone number, patients could not be contacted for follow-up which was reflected by low participant rates.

There was significant difficulty getting participants’ agreement to a home visit by EMS based on the EMS telephone contact effort. Several factors that contributed to this were failure to self-identify as at-risk, currently receiving care at the time of phone call (including hospitalization), unable to contact, and no interest in speaking with a representative from EMS. It was also difficult to find one time frame (e.g., 1-week post initial EMS call) that could be applied to all patients to call to schedule a follow-up. To address these barriers, patients are now to be asked at the time of the field assessment if they would like a follow-up and contact information will be obtained for both the patient and their primary caregiver (if possible). This process amendment will hopefully reduce the difficulty in trying to explain the program over the phone and make it easier to schedule a follow-up visit.

The 3-month follow-up survey revealed areas of success and room for improvement. One participant remarked that the, “EMS made suggestions to get the wheelchair through the doorway” while a family member of another participant commented, “The older adult won’t comply with the recommendations.” Some of the additional comments included: (1) “Daughter was very frustrated – she has been spending considerable time caring for her mother and needs help. The daughter has minimal transportation and hasn’t been able to go to work due to caring for her mother. Her mother had been taken to the hospital in the morning with a mini-stroke. The daughter repeatedly said that her mother needs a wheelchair,” (2) “Talked with son-in-law of patient. He was present at the EMS home visit and very enthusiastically supports it.”

The idea of utilizing EMS to provide population health services is not novel; programs have been established for the EMS staff to augment immunization and fall prevention services provided in the rural areas of upstate New York (10). SUAA successfully expands this model of care beyond a rural setting. In addition, SUAA partners with the Department of Aging in order to bolster program recruitment and to offer evaluation and meaningful interventions in the care of falls risk patients. The follow-up rate for the study in upstate New York was 61% with the survey completed 14 days after interview; follow-up calls were attempted for up to 4 weeks to contact individuals (10). The 3-month wait time for SUAA allowed adequate time for all planned interventions to be performed prior to assessing the patient outcomes. Still, the SUAA respondent rates are lower than NY study, and further studies looking into barriers to communication may be warranted.

The study shows SUAA addresses a need within the community, but adjustments are needed to improve processes to ensure sustainability.

## Conclusion

Elderly, frail patients with multi-morbidity require greater time and resources to maintain independent living. In an effort to intercept the unique health-care concerns of a rapidly expanding aging population, the SUAA offers a potential solution by targeting at-risk individuals and providing assessment and resources. The goal is to not only decrease the number of EMS calls for falls but also the overall community morbidity as a result of preventable falls in older adults. This program represents a tremendous effort put forth by UNC, OCDoA, and OCES. The historical data and results from the pilot phase of Stay Up and Active demonstrate the need in Orange County for more than simple emergency response to injury and illness. Orange County EMS is in a prime position to provide the falls assessment questions as an integrated part of their services, and must continue implementation of this program as well as address the barriers identified in this report. Furthermore, SUAA represents a national trend for EMS systems to address community needs of their patients and begin to shift resources toward population health as a means to alleviate the burdens they face. However, with a large aging population, both local and national attention should be given to help individuals safely age in place as a way to help offset future health-care costs.

## Author Contributions

SL is the primary author for this work. LE served as content and conceptual review for the work throughout the process. TS was the program implementation lead. KK made iterative changes and periodic updates to the work throughout the process. ER and JB-W provided subject-matter expertise to the research and evaluation oversight.

## Conflict of Interest Statement

The authors declare that the research was conducted in the absence of any commercial or financial relationships that could be construed as a potential conflict of interest.

## Abbreviations

AGS, The American Geriatrics Society; ALS, advanced life support; CDC, Centers for Disease Control and Prevention; C-Spine, cervical spine; ED, emergency department; EMS, emergency medical services; EMT, emergency medical technician; OCDoA, Orange County Department on Aging; OCES, Orange County Emergency Services; STEADI, stopping elderly accidents, deaths, and injuries; SUAA, stay up and active; TBI, traumatic brain injury; UNC, University of North Carolina at Chapel Hill.
